# Subclinical cognitive deficits are associated with reduced cerebrovascular response to visual stimulation in mid-sixties men

**DOI:** 10.1007/s11357-022-00596-2

**Published:** 2022-06-01

**Authors:** Mark Bitsch Vestergaard, Ulrich Lindberg, Maria Højberg Knudsen, Olalla Urdanibia-Centelles, Aftab Bakhtiari, Erik Lykke Mortensen, Merete Osler, Birgitte Fagerlund, Krisztina Benedek, Martin Lauritzen, Henrik Bo Wiberg Larsson

**Affiliations:** 1 Functional Imaging Unit, Department of Clinical Physiology, Nuclear Medicine, and PET, Copenhagen University Hospital Rigshospitalet, Valdemar Hansens Vej 1-23, 2600 Glostrup, Denmark; 2 Department of Clinical Neurophysiology, Copenhagen University Hospital Rigshospitalet, Glostrup, Denmark; 3 Department of Public Health, University of Copenhagen, Copenhagen, Denmark; 4 Child and Adolescent Mental Health Center, Copenhagen University Hospital, Mental Health Services CPH, Copenhagen, Denmark; 5 Faculty of Social Sciences, Department of Psychology, University of Copenhagen, Copenhagen, Denmark; 6 Faculty of Health and Medical Science, Department of Clinical Medicine, University of Copenhagen, Copenhagen, Denmark

**Keywords:** Cerebrovascular function, Brain physiology, Cerebral perfusion, Neurovascular coupling, Brain ageing

## Abstract

Reduced cerebrovascular response to neuronal activation is observed in patients with neurodegenerative disease. In the present study, we examined the correlation between reduced cerebrovascular response to visual activation (ΔCBF_Vis.Act_) and subclinical cognitive deficits in a human population of mid-sixties individuals without neurodegenerative disease. Such a correlation would suggest that impaired cerebrovascular function occurs before overt neurodegenerative disease. A total of 187 subjects (age 64–67 years) of the Metropolit Danish Male Birth Cohort participated in the study. ΔCBF_Vis.Act_ was measured using arterial spin labelling (ASL) MRI. ΔCBF_Vis.Act_ correlated positively with cognitive performance in: Global cognition (*p* = 0.046), paired associative memory (*p* = 0.025), spatial recognition (*p* = 0.026), planning (*p* = 0.016), simple processing speed (*p* < 0.01), and with highly significant correlations with current intelligence (*p* < 10^−5^), and more complex processing speed (*p* < 10^−3^), the latter two explaining approximately 11–13% of the variance. Reduced ΔCBF_Vis.Act_ was independent of brain atrophy. Our findings suggest that inhibited cerebrovascular response to neuronal activation is an early deficit in the ageing brain and associated with subclinical cognitive deficits. Cerebrovascular dysfunction could be an early sign of a trajectory pointing towards the development of neurodegenerative disease. Future efforts should elucidate if maintenance of a healthy cerebrovascular function can protect against the development of dementia.

**Supplementary Information:**

The online version contains supplementary material available at 10.1007/s11357-022-00596-2.

## Introduction

Cerebrovascular dysfunction is observed in neurodegenerative disease, most notably in vascular dementia (VD) but also in Alzheimer’s disease (AD) [1, 2]. Patients with AD demonstrate reduced neurovascular coupling (NVC) [3] and reduced cerebral blood flow (CBF) and glucose metabolism in response to neuroactivation tasks [4, 5]. Similarly, patients with mild cognitive impairment (MCI) demonstrate reduced cerebrovascular response evoked by memory tasks [6, 7]. Global cerebrovascular reactivity (CVR) challenged by breath holding or inhalation of CO_2_ is also significantly reduced in both dementia and MCI patients [1, 8, 9]. These vascular dysfunctions are seen in early AD disease stages, suggesting that these dysfunctions could be a contributing factor in the development of neurodegenerative disease [10, 11]. Cerebrovascular dysfunction also appears to interact with other AD pathologies, thus creating a harmful positive-feedback loop that aggravates disease progression [12, 13]. For example, impaired cerebrovascular function will inhibit clearance of beta amyloid (Aβ) or tau protein tangles [14], and the accumulated proteins then further exacerbate vascular dysfunction [11].

Elevated CBF in response to neuronal activation shares similarities with the increase in CBF from exposure to CO_2_ but works through different pathways. Both mechanisms rely on cerebral vasodilation governed by vascular smooth muscle cells (VSMC) and pericytes covering the arteries and capillaries, respectively. During neuronal activation, the CBF increases in the affected regions through the NVC. The NVC works as a feedforward mechanism by the release of vasodilating agents when the neurons are activated causing local vasodilation and increased perfusion [15, 16]. The increase in CBF from CO_2_ exposure is a result of vasodilation caused by direct relaxation of VSMC from CO_2_ accumulation [17].

The aim of the present study was to examine whether reduced cerebrovascular function can be related to subclinical cognition deficits in a cohort of mid-sixties adults without neurodegenerative disease. We examined both the local increase in CBF from neuroactivation and the global increase in CBF in response to CO_2_ exposure from a breath hold challenge. Using this setup, we obtained information on the cerebrovascular function both through the NVC mechanism and through direct stimulation of the VSMC. A correlation between impaired cerebrovascular function and cognitive deficits could indicate a very early deficit in the ageing brain and therefore may be an important causal or contributing factor in the development of neurodegenerative disease.

## Methods

### Participants

A total of 187 subjects participated in this study. The participants were enrolled as part of the Metropolit Danish Male Birth Cohort [18, 19], which includes males born in 1953 in a Copenhagen metropolitan area. The cohort includes data concerning socioeconomic factors, health, and cognitive status from youth until today. From the Danish military draft system, all Danish men undergo draft examination at approximately 18–20 years of age. The cohort utilizes this system by obtaining measurements of cognitive function from the draft examination. The participants additionally underwent cognitive testing at age ~ 57 years as part of the Copenhagen Ageing and Midlife Biobank project (CAMB) study [19]. At age 64–67, all participants underwent extensive cognitive examination and participated in one magnetic resonance imaging (MRI) scan from which the brain physiology data presented in this study were acquired. The MRI scans were performed from January 2018 until March 2020. Cerebrovascular function in response to breath holding and measurements of lactate using MR spectroscopy (MRS) were only performed in a subset of the participants (*n* = 85 for breath holding challenge and *n* = 126 for MRS acquisition).

Studies based on the Metropolit Danish Male Birth Cohort on age-related changes in brain structures [20, 21] and electrical activity [22, 23] and its relationship to cognition have previously been published; however, the participants in present study were not part of the former studies.

The study was approved by the Capital Region of Denmark’s Committee on Health Research Ethics (2014–41-2998, 2008–41-2938 and H-1–2014-032).

### MRI protocol

All MRI scans were acquired on a Philips 3 T dSTREAM Achieva MRI scanner (Philips Medical Systems, Best, The Netherlands) using a 32-channel phased array head coil. To examine cerebrovascular function, we measured the increase in cerebral perfusion in the visual cortex in response to visual stimulation (ΔCBF_Vis.Act_) acquired using arterial spin labelling (ASL) MRI, and we recorded the global cerebrovascular reactivity (CVR) in response to breath holding using phase contrast mapping (PCM) MRI. To obtain a metabolic response to neuroactivation, we additionally measured the increase in cerebral lactate concentration from visual activation using MR spectroscopy (MRS). Resting global CBF (gCBF) and resting global cerebral metabolic rate of oxygen (gCMRO_2_) were acquired using PCM MRI and susceptibility-based oximetry (SBO) MRI techniques. Brain atrophy was assessed by structural MRI.

#### Cerebrovascular response to neuroactivation

**Fig. 1:**
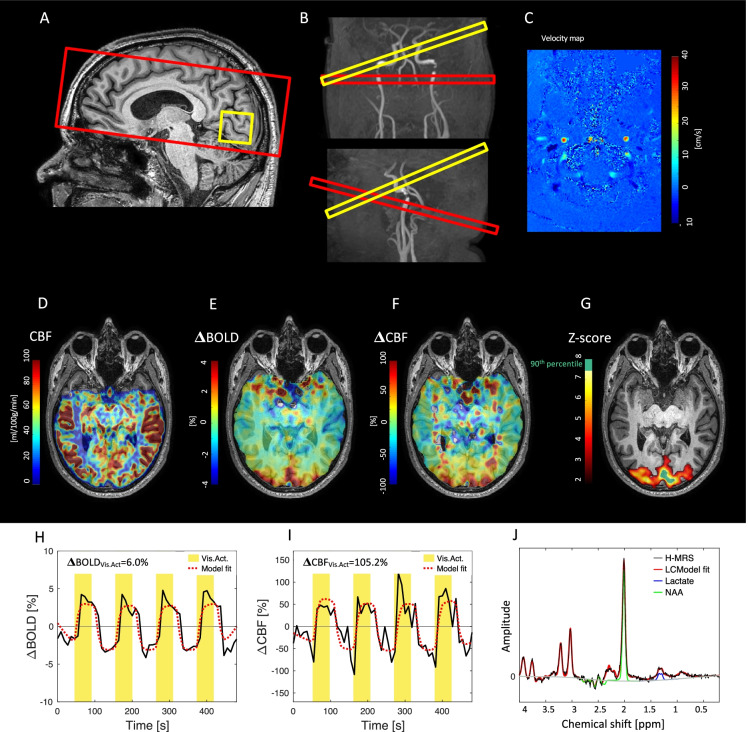
Examples of data and processing of MRI data to measure the cerebrovascular response to neuroactivation (ΔCBF_Vis.Act_), global cerebral blood flow (gCBF), and lactate concentration. The figure demonstrates the analysis from a single representative subject. (**A**) The red box demonstrates the field of view for the acquisition of the dual-echo pseudocontinuous arterial spin labelling (pCASL) sequence from which ΔCBF_Vis.Act_ could be assessed. The yellow box demonstrates the location of the MRS voxel placed in the visual cortex to measure lactate concentration. (**B**) Imaging planes located perpendicular to the carotid arteries (red box) and basilar artery (yellow box) of the phase-contrast mapping (PCM) sequence for acquisition of quantitative gCBF are shown superimposed on an arterial MRI angiogram. (**C**) From the PCM sequence, a velocity map can be acquired from which the gCBF can be calculated. From the dual-echo pCASL sequence, cerebral blood flow (CBF) maps (**D**) were acquired in addition to BOLD-weighted maps. For this visualization, the CBF maps were normalized to the gCBF value acquired by PCM MRI. Voxel-wise general linear regression models with the block stimulation paradigm as the regressor were used to locate the brain areas activated from the visual stimulation. Panels **F** and **G** demonstrate maps of regression coefficients for the voxel-vise regression modelling of the BOLD and CBF signals, respectively. Higher regression coefficients in the visual cortex are clearly visible demonstrating activation in this area. Z-score statistics were used to locate the area with the most significant activation (**G**). (**H**) The change in BOLD-signal (ΔBOLD_Vis.Act_) and (**I**) CBF (ΔCBF_Vis.Act_) in the most activated region of the visual cortex (above the 90th percentile z-score) was found and used in the further analysis. The lactate and N-acetylaspartate (NAA) concentrations in the visual cortex were measured using MR spectroscopy (J). A long echo time (288 ms) was used to measure the spectrum to ensure a clear lactate peak in the spectrum

Cerebrovascular response to neuroactivation (ΔCBF_Vis.Act_) was measured using an MRI-sequence from which both blood-oxygen-level-dependent (BOLD)-weighted and CBF-weighted images could be acquired. An example of the analysis from a single representative subject is shown in Fig. 1. A 2D gradient-echo dual-echo pseudocontinuous arterial spin labelling (pCASL) sequence with echo-planar imaging (EPI) as readout was used (16 slices, FOV = 240 × 140 × 95 mm^3^; acquired voxel size = 2.75 × 2.75 × 5 mm^3^; reconstructed voxel size = 1.875 × 1.875 × 5 mm^3^; TR = 4550 ms; TE_1_ = 13 ms; TE_2_ = 31.7 ms; flip angle = 90°; 54 dynamics, total duration = 8 min 12 s; SENSE factor = 2.3). Pseudo-continuous labelling scheme (label distance = 90 mm; label duration = 1800 ms; post label delay = 1800 ms) was used for arterial spin labelling to obtain blood-labelled images. CBF maps were calculated by subtracting blood-labelled and nonlabelled control images. An example of an acquired CBF image is shown in Fig. 1D. BOLD-weighted maps were acquired by using the nonlabelled images acquired from the second echo (TE = 31.7 ms). Both the BOLD and CBF maps were smoothed by a Gaussian 5-mm filter. The time series in each voxel were high-pass filtered with a 90 s cut off.

The subjects were visually stimulated by a flickering black and white checkerboard shown in MRI-compatible goggles or at a screen in the MRI scanner. The flickering checkerboard was presented four times for 54 s each, with a black screen also lasting 54 s before, after, and in-between each stimulation block. We used stimulation at 8 Hz, which resulted in the highest metabolic response in a study using similar MRI techniques [24].

The activated brain regions from visual stimulation were calculated by modelling the BOLD maps by voxel-wise general linear regression models with the stimulation paradigm as the regressor using FSL Feat [25]. The regressor was created by convoluting the block paradigm with the hemodynamic response function modelled by a gamma distribution function (mean lag = 6 s, SD = 3 s). Before entering the modelling, the BOLD signals were normalized to a mean of zero. The regression coefficients from the models describe the average change in BOLD signal from the four blocks of visual stimulation in percentage. In Fig. 1E, an example of a map showing the regression coefficients from the voxel-wise linear regression models is shown. High values demonstrate activated areas. In each voxel, the z-score statistic from the regression model was calculated to generate z-score maps. The significantly activated areas were found by defining the voxel on the z-score map with values above 3.1. An example of a z-score map is shown in Fig. 1G. The largest contiguous activated region was located, and in this area, the voxel in the upper 10 percentile of z-scores was used to generate a region of interest (ROI) describing the brain region with maximal activation. The BOLD change in this ROI (ΔBOLD_Vis.Act_) was calculated as the mean of the regression coefficients from the linear model in all voxels in the ROI. The ROI was hereafter applied to the CBF maps, and the median time series of the voxels in the ROI were calculated. The CBF time series were normalized to a mean of zero and then modelled by a general linear regression model with the block paradigm of stimulation as the regressor, which was similar to the model used to locate the activated brain regions. The regression coefficient from this model describes the average change in CBF from the four blocks of visual stimulation in percentage (ΔCBF_Vis.Act_). Examples of the time series of change in BOLD and CBF signals in the most activated area, from which ΔBOLD_Vis.Act_ and ΔCBF_Vis.Act_ are derived, are demonstrated in Fig. 1H–I.

Measurements of quantitative CBF changes from neuroactivation using ASL MRI technique have been validated against accepted reference standard [^15^O]H_2_O positron emission tomography (PET) imaging [26] and have a good reproducibility [27].

#### Global cerebral blood flow

The gCBF was calculated by measuring the blood flow through the feeding cerebral arteries using velocity-sensitive phase contrast mapping (PCM) MRI [28, 29]. In neurodegenerative disease, gCBF will be reduced. Using PCM MRI, blood velocity maps was acquired by a velocity-encoding turbo field echo sequence (1 slice, FOV = 240 × 240 mm^2^; voxel size = 0.75 × 0.75 × 8 mm^3^; TE = 7.33 ms; TR = 27.63 ms; flip angle = 10°; velocity encoding = 100 cm/s, without cardiac gating) (Fig. 1C).

Two separate measurements were acquired: first, an imaging plane perpendicular to the carotid arteries and a second measurement perpendicular to the basilar artery (Fig. 1B). The blood flows in the cerebral arteries were calculated by multiplying the mean blood velocity by the cross-sectional area from regions of interest (ROIs) defining each vessel. By normalizing the total cerebral blood flow to the brain weight, gCBF was calculated. Brain weight was estimated from the segmentation of the structural MRI image assuming a brain density of 1.05 g/ml [30].

The use of PCM to acquire gCBF has been validated in pigs and humans against ^15^O-water PET imaging as an accepted standard reference [29, 31, 32].

#### Cerebral metabolic rate of oxygen

By SBO MRI technique, the venous oxygen saturation of the blood leaving the brain in the sagittal sinus was acquired [33, 34]. The SBO technique utilizes that the difference in magnetic susceptibility between deoxyhemoglobin in venous blood and the surrounding tissue can be related to venous oxygen saturation [35]. Susceptibility-weighted maps were calculated by acquiring phase maps from two different echo times using a dual-echo gradient-echo sequence and subtracting the phase maps from each echo (1 slice, FOV = 220 × 190 mm^2^; voxel size = 0.5 × 0.5 × 8 mm^3^; TE_1_ = 10.89 ms; TE_2_ = 24.16 ms; flip angle = 30°; 5 repeated measures, total duration = 1 min 30 s; SENSE-factor = 2). The imaging plane was placed perpendicular to the sagittal sinus. Aliased phase values in the sagittal sinus and immediately surrounding tissue were manually corrected. The sequence and postprocessing have been previously described in-depth [36, 37]. Measurements of oxygen saturation in the sagittal sinus by SBO have been validated against blood samples acquired by catheter in vena jugularis concurrently with MRI scanning [38]. Validation was performed both at rest and during hyperperfused conditions.

In combination with the gCBF measurement, gCMRO_2_ could be calculated using the Fick principle (Eq. 1).

1 $${\mathrm{gCMRO}}_{2}=[\mathrm{Hgb}]\bullet \mathrm{gCBF}\bullet ({\mathrm{S}}_{\mathrm{a}}{\mathrm{O}}_{2}-{\mathrm{S}}_{\mathrm{v}}{\mathrm{O}}_{2})$$

The hemoglobin concentration was measured from venous blood samples, and arterial oxygen saturation (S_a_O_2_) was measured using pulse oximetry.

#### Cerebrovascular reactivity by breath holding

**Fig. 2:**
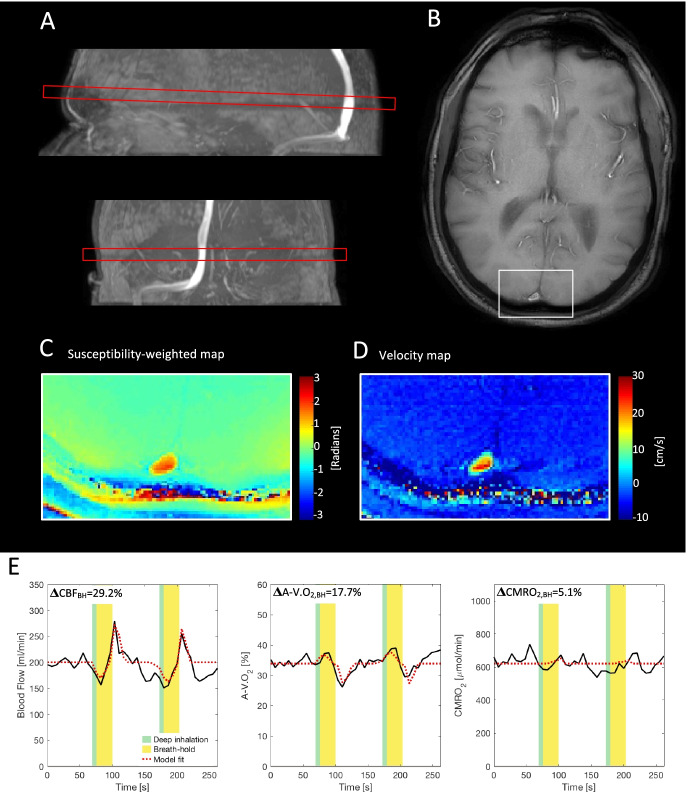
Example of data acquired for calculating the cerebral blood flow (CBF), arteriovenous oxygen saturation difference (A-V.O_2_), and metabolic rate of oxygen (CMRO_2_) in response to breath holding. The figure demonstrates the analysis from a single representative subject. (**A**) Sequence combining phase contrast mapping (PCM) and susceptibility-based oximetry (SBO) for measuring flow and oxygen saturation was acquired perpendicular to the sagittal sinus. By examining the intravascular voxels in the sagittal sinus and the immediately surrounding area (**B**), oxygen saturation can be acquired from a susceptibility-weighted map (**C**), and blood flow can be calculated from the velocity-weighted map (**D**). The change in blood flow, A-V.O_2_, and CMRO_2_ derived using Fick’s principle are calculated through a paradigm including two breath holds of 36 s. Examples of time series of blood flow, A-V.O_2_, and CMRO_2_ along with indication of the breath-hold paradigm are shown in panel (**E**). The regressors (black line) and fit (red line) from the linear regression models are additionally shown in panel (**E**). For this subject, the average peak increase in CBF during the two breath-holds was 29.2%. Correspondingly, A-V.O_2_ decreased with on average 17.7% and CMRO_2_ was 5.1% higher at the peak of the breath-hold

In order to measure the dynamic changes of CBF and CMRO_2_ through a breath hold challenge, an MRI sequence combining PCM and SBO techniques was used [39]. An example of the analysis in a single representative subject is demonstrated in Fig. 2. By using a sequence combining PCM and SBO, the blood flow and oxygen saturation of the blood leaving the brain in the sagittal sinus could be acquired simultaneously. A dual-echo gradient-echo sequence with turbo field echo readout was used (1 slice, FOV = 220 × 190 mm^2^; voxel size = 0.688 × 0.688 × 8 mm^3^; TE_1_ = 8.02 ms; TE_2_ = 17.72 ms; flip angle = 30°; 39 repeated measures, total duration = 4 min 36 s; SENSE-factor = 2). A velocity encoding phase contrast-mapping scheme was added to the sequence to calculate a velocity-weighted map (VENC = 100 cm/s, without cardiac gating). An example of an acquired velocity map is demonstrated in Fig. 2D. The breath-hold paradigm consisted of the two breath holds lasting 36 s, with a 50-s break between each breath hold. Initial baseline values were acquired for 50 s before the first breath hold (Fig. 2E). Instructions describing when to start and end breath holding were presented by text on a screen to the subjects in the MRI scanner.

The blood flow in the sagittal sinus (BF_ss_) through the breath holds were calculated by multiplying the mean blood velocity by the cross-sectional ROI defining the sagittal sinus, which was similar to the analysis of gCBF described earlier. Venous oxygen saturation was found by calculating susceptibility-weighted maps from the two echoes and relating the differences between intravenous values and surrounding tissue values, which was similar to the calculation of the CMRO_2_ described earlier. An example of a susceptibility-weighted map is shown in Fig. 2C. The CMRO_2_ through the breath holds was calculated by Fick’s principle using BF_ss_. An in-depth description of the method for measuring blood flow and CMRO_2_ during breath holding has been previously provided [37].

The change in blood flow and CMRO_2_ from breath holding was calculated by modelling the time series by a general linear regression model. The overall mean changes from breath holding from all subjects were used as the regressor. The regressor was normalized to the largest response in the breath-hold scheme. The time series were normalized by subtracting the baseline values before the first breath hold before entering the linear regression. The regression coefficient from the model will therefore describe the percentage change from baseline values before breath holding (ΔCBF_BH_ and ΔCMRO_2_,_BH_). Examples of time series of blood flow, arteriovenous oxygen saturation difference (A-V.O_2_), and CMRO_2_ throughout the breath hold paradigm and the corresponding fits from the linear regression models are shown in Fig. 2E.

#### MR spectroscopy

Lactate and N-acetylaspartate (NAA) concentrations were obtained by MR spectroscopy using a water-suppressed point-resolved spectroscopy (PRESS) pulse sequence (TR = 3000 ms; TE = 288 ms; voxel size = 30 × 35 × 30 mm^3^, water suppression) (Fig. 1J). The sequence was optimized to measure lactate by using a long echo time of 288 ms. The MRS voxel was placed in the visual cortex (Fig. 1A).

The NAA concentration is a marker related to neuronal integrity and function. NAA is reduced in patients with neurodegenerative disease [40]. NAA was measured to test for the correlation with cognitive performance. A correlation between reduced NAA and poor cognitive performance would suggest a beginning neurodegeneration.

Lactate concentrations were obtained to examine a metabolic response from neuroactivation. Two measurements were obtained: one measurement during rest and a second measurement during continuous visual stimulation by an 8 Hz flickering checkerboard, which was similar to the stimulation used to assess the cerebrovascular response. In the healthy brain, the lactate concentration increases in activated brain regions by approximately 5–30% [41].

#### Structural images

Structural brain images were acquired by a 3D T1-weighted turbo field echo sequence (echo time (TE) = 5.11 ms; repetition time (TR) = 11.2 ms; flip angle = 8°, field of view (FOV) = 240 × 256 × 180 mm^3^; voxel size = 0.70 × 0.76 × 0.70 mm^3^).

The images were segmented using the FreeSurfer software package (v5.3.0, Martinos Center for Biomedical Imaging, Massachusetts, USA) and analyzed using standard and recommended procedures [42, 43]. The brain was segmented into 34 cortical brain regions in each hemisphere and 13 substructures (left and right amygdala, hippocampus, pallidum, putamen, caudate, thalamus, and brainstem) based on the Desikan-Killiany Atlas [44]. The volume of each segmented region and the cortical thickness of the cortex regions were obtained. Furthermore, the intracranial volume (ICV) was found, and the global brain volume was calculated by mask including the hemisphere, cerebellum, and the brainstem but excluding the ventricles and cerebrospinal fluid (CSF).

### Cognition

Assessment of the subjects’ cognition was performed prior to MRI scanning. Intelligence and global cognitive function were assessed using the Intelligenz-Struktur-Test 2000R (IST 2000R) and Addenbrooke’s cognitive examination (ACE). Processing speed was tested using Trail Making Tests A and B and the Symbol Digit Modalities Test (SDMT). Paired associate memory functions were assessed using 50 word pairs learning and recall and the Paired Associates Learning Task (PAL) test from the Cambridge Neuropsychological Test Automated Battery (CANTAB). Pattern and spatial recognition were assessed by the Pattern Recognition Memory test (PRM) and the Spatial Recognition Memory test (SRM) and spatial planning using the Stockings of Cambridge test (SOC) from CANTAB.

Additionally, the subjects were tested using the IST 2000R at age ~ 57 years and the Børge Priens Prøve (BPP) at age ~ 20 years. From these earlier tests, the longitudinal change in intelligence (ΔIQ._65–20_ and ΔIQ._65–57_) could be addressed. ΔIQ._65–20_ ΔIQ._65–57_ were calculated by subtracting the measurement at age 20 and 57 from the IST 2000R test score acquired at the time of MRI scanning (age ~ 65). Before subtracting the values, the measurements were normalized to mean zero and unit standard deviation.

### Health status

To examine whether health parameters and lifestyle factors could explain differences in cerebrovascular function, the following health parameters were collected: history of brain disease, cardiovascular disease, cancer, or psychiatric disease, hypertension (yes/no), diabetes mellitus (yes/no), hypercholesterolemia (yes/no), hyperlipidemia (yes/no), body mass index (BMI), smoking history (current usage and pack years), alcohol usage (alcohol units per week), physical activity (exercise frequency), and education attainment (number of school years). The health parameters were self-reported, except BMI which were measured at the time of participation. The average values of the health parameters are presented in Table 1, and histograms demonstrating the distributions can be seen in supplementary information (Figure S1).

**Table 1:** Summary of health parameters and lifestyle factors. For categorical measurements, the amounts and percentages are noted. For continuous measurements, the mean and 95% confidence interval (CI) are noted. Histograms of the parameters are demonstrated in the supplementary information (Figure S1)

Health parameter	Value
Age (years)	65.7 (CI: 64.6–66.8)
Exercise frequency	
Daily	31 (16.6%)
2–3 per week	83 (44.4%)
1 per week	26 (13.9%)
2–3 per month	3 (1.6%)
Never	38 (20.3%)
Not reported	6 (3.2%)
Body mass index (kg/cm^2^)	27.5 (CI: 21.3–35.8)
Alcohol usage (units of alcohol per week)	10.5 (CI: 0–35.0)
Smoking history (pack years)	9.7 (CI: 0–42.0)
Current smoker (yes/no)	24 (12.8%)/163 (87.2%)
Hypertension (yes/no)	72 (39.6%)/113 (60.4%)
Hyperlipidemia (yes/no)	8 (4.2%)/179 (95.7%)
Hypercholesterolemia (yes/no)	51 (27.3%)/136 (72.7%)
Diabetes (yes/no)	13 (7.0%)/174 (93.0%)
History of heart disease (yes/no)	33 (17.6%)/154 (82.4%)
History of stroke (yes/no)	10 (5.3%)/177 (94.7%)
Education attainment (school years)	11.0 (CI: 8–14)

### Statistics

#### Cerebrovascular function and cognition

Correlations between cognitive parameters and cerebrovascular response to activation (ΔCBF_Vis.Act_ and ΔBOLD_Vis.Act_) and between cognition and cerebrovascular reactivity from breath holding (ΔCBF_BH_ and ΔCMRO_2,BH_) were calculated by general linear regression models (Table 2). Post hoc analyses with exercise frequency or BMI as additional regressor were performed to examine if exercise habits could explain a possible correlation between cognition and cerebrovascular function, as prior studies have demonstrated correlations between cardiovascular fitness and both cognition and cerebrovascular reactivity [45, 46]. Correlations between cognition parameters and resting physiology were tested by a general linear regression model with CBF, CMRO_2_, and NAA as regressors and the cognition parameters as the dependent variable (see supplementary information Table S1).

*P*-values from the correlations were calculated both without and with correction for multiple comparisons using the false discovery rate (FDR) (*q* = 0.05).

The correlation between ΔCBF_Vis.Act_ and ΔCBF_BH_ was calculated using a general linear regression model to test for agreement in cerebrovascular response between the two stimuli.

The correlation between ΔCBF_Vis.Act_ and ΔLac_Vis.Act_ was calculated using a general linear regression model to test if reduced cerebrovascular response affected the metabolic response to neuroactivation.

Robust fitting was used in the regression models.

#### Cerebrovascular function and brain atrophy

To assess the correlation between reduced cerebrovascular response and brain atrophy, we calculated general linear regression models with ΔCBF_Vis.Act_ or ΔCBF_BH_ as the regressors and each brain region from the segmentation of the brain as dependent variables. The brain volumes were normalized to intracranial volume (ICV) before entering the regression models. Correlations were corrected for multiple comparisons using FDR (*q* = 0.05). Additionally, surface-based analysis correlating cortical thickness to ΔCBF_Vis.Act_ or ΔCBF_BH_ was performed using group comparison functions from the FreeSurfer software package [47]. Each vertex describing cortical thickness from the FreeSurfer segmentation was correlated to ΔCBF_Vis.Act_ or ΔCBF_BH_ by general linear regression models and corrected for multiple comparisons by a clusterwise correction using permutation simulation [47].

#### Cerebrovascular function and health status

General linear regression models were calculated to examine whether ΔCBF_Vis.Act_ or ΔCBF_BH_ was correlated with health parameters and lifestyle factors using a model that included history of heart disease, history of stroke, hypertension, diabetes, hypercholesterolemia, BMI, smoking history (pack years), alcohol per week, exercise activity, and education as regressors. Exercise activity entered as a categorical variable divided by the activity at least once a week. A history of stroke was not included in the model of ΔCBF_BH_ because only one subject in this subgroup had a history of stroke.

Post hoc stepwise linear regression models were performed to reduce the model to discover the health parameters that best describe the potential reduced cerebrovascular function. The R^2^ value was used to find the optimal model.

## Results

### Attrition and missing data

Anatomical images and measurements of gCBF were completed in all participants. In six subjects, CMRO_2_ was not measured. In three of these subjects, the MRI sequence for measuring venous oxygen saturation was not available due to technical issues, and for the remaining three subjects, the measurement of venous saturation was not feasible due to bifurcation of the sagittal sinus [33].

In four subjects, ΔCBF_Vis.Act_ was not measured due to technical issues, making the MRI sequence or the visual stimulation system unavailable at the time of MRI acquisition. In 22 participants, we could not measure an activation pattern from visual stimulation in the visual cortex. In addition, in 9 subjects, we did not obtain a significant CBF response from the ASL measurements in the activated area. These measurements were excluded from the analysis. One subject demonstrated a physiologically anomalous CBF response to visual stimulation (> 400%) and was removed from the analysis. In 16 of these measurements, the lack of activation pattern or significant ASL response could be related to motion artefacts. One subject did not complete the full MRI session, and acquisition of ASL response was not completed. In total, 151 measurements of cerebrovascular response to neuroactivation were completed and included in the analyses.

One subject could not complete the breath hold challenge, and this measurement was excluded from the analyses.

### Cerebrovascular response to neuroactivation

**Fig. 3:**
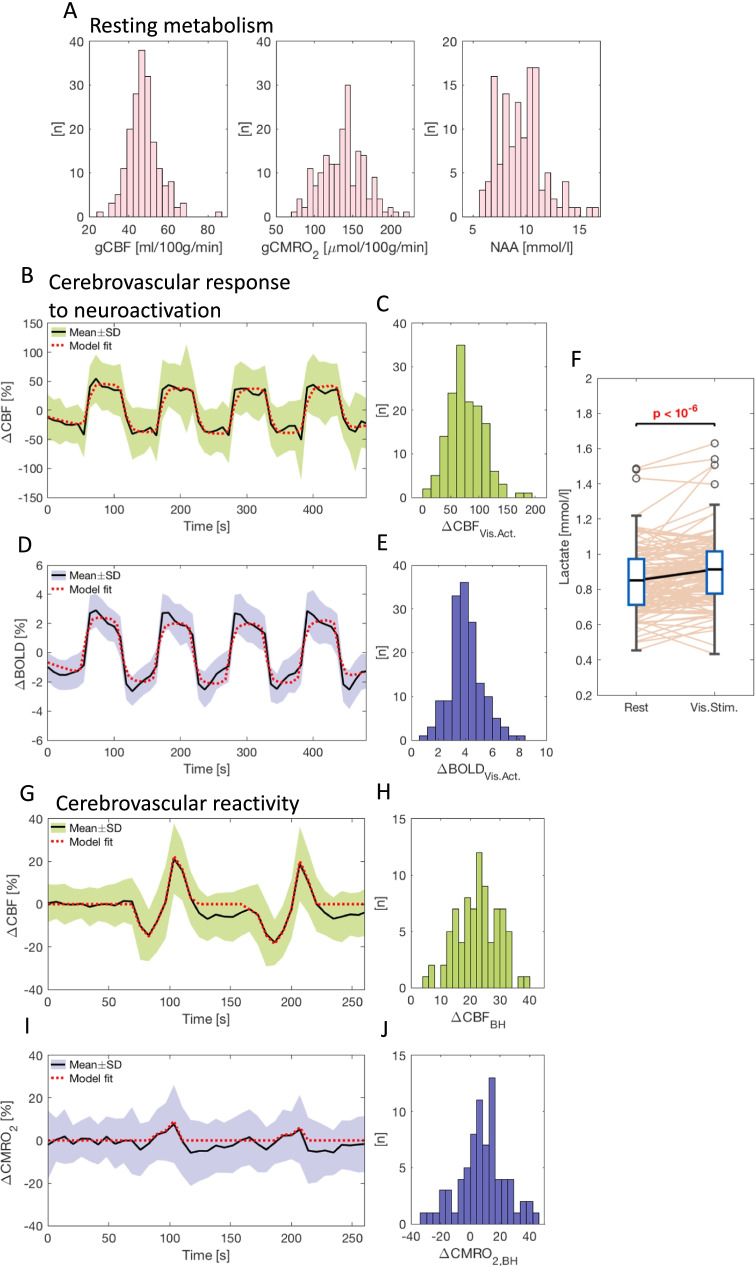
Visualization of the acquired cerebrovascular response to neuroactivation, cerebrovascular reactivity, and resting metabolism parameters. Distributions of global cerebral blood flow (gCBF), metabolic rate of oxygen (gCMRO_2_) and n-acetylaspartate (NAA) concentration are demonstrated in panel **A**. Mean time series of change in CBF and BOLD signal through the neuroactivation paradigm are shown in panels **B** and **D**, and the corresponding distribution of ΔCBF_Vis.Act_ and ΔBOLD_Vis.Act_ acquired from the linear regression model fits are shown in panels **C** and **E**. Mean time series of change in blood flow and CMRO_2_ throughout a paradigm of two breath holds of 36 s are presented in panels **G** and **I**, and the corresponding distribution ΔCBF_BH_ and ΔCMRO_2,BH_ acquired from the regression model fits are presented in panels **H** and **J**. The lactate concentration at rest and during neuroactivation is shown in panel **F**

Neuroactivation by visual stimulation caused, on average, a 77.1 ± 30.5% increase in CBF (ΔCBF_Vis.Act_) across the studied population (Fig. 3B–C).

**Fig. 4:**
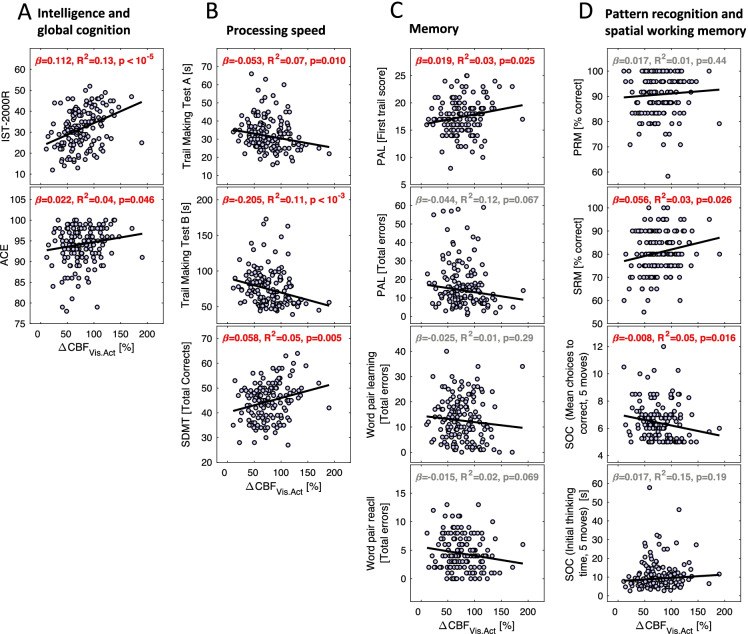
Correlations between cerebrovascular response to neuroactivation (ΔCBF_Vis.Act_) and results from the cognition tests. The cognition tests can be divided into four cognitive domains: general cognition (**A**), processing speed (**B**), memory (**C**), and pattern recognition and spatial working memory (**D**). Low ΔCBF_Vis.Act_ correlated significantly with poor performance of the IST-2000R, ACE, Trail Making Test A and B, SDMT, PAL first trail score, SRM, and SOC. Abbreviations: IST 2000R, Intelligenz-Struktur-Test 2000R; ACE, Addenbrooke’s Cognitive Examination; SDMT, Symbol Digit Modalities Test; PAL, paired associates learning; SRM, spatial recognition memory; SOC, Stocking of Cambridge

To test whether the cerebrovascular response to neuroactivation was related to cognitive functions, ΔCBF_Vis.Act_ was correlated with performance on several cognitive measures (Table 2, Fig. 4). ΔCBF_Vis.Act_ correlated significantly and positively with poor performance in multiple cognitive tests (IST 2000R, ACE, Trail Making Test A and B, SDMT, PAL (first trial score), SRM, and SOC (mean moves)). If the *p*-values were corrected for multiple comparisons, the correlations remained significant (*p* < 0.048), except for the correlation with ACE (*p* = 0.075). The most highly significant and strongest correlations (explaining approximately 11–13% of the variance) were seen with current intelligence measured on the IST 2000R, and processing speed measured on the Trail Making B. Models with exercise frequency or BMI included as regressor did not substantially alter the significant correlations between ΔCBF_Vis.Act_ and cognition (not shown), indicating that exercise habits could not explain the relation between cognition and cerebrovascular function.

**Table 2:** Summary of correlations between cognition and ΔCBF_Vis.Act_, ΔBOLD_Vis.Act_ and ΔCBF_BH_. *A statistically significant change (*p* < 0.05). Abbreviations: *IST 2000R*, Intelligenz-Struktur-Test 2000R; *ACE*, Addenbrooke’s Cognitive Examination; *SDMT*, Symbol Digit Modalities Test; *PAL*, paired associates learning; *RVP*, rapid visual information processing; *SRM*, spatial recognition memory; *SOC*, Stocking of Cambridge

	Neuroactivation						Breath holding		
	ΔCBF_Vis.Act_			ΔBOLD_Vis.Act_			ΔCBF_BH_		
Cognition domain	*β*	R^2^	*p*	*β*	R^2^	*p*	*β*	R^2^	*p*
**Intelligence and global cognition**									
IST 2000R	0.112	0.13	< 10^−5^*	1.805	0.05	0.005*	0.370	0.08	0.008*
ACE	0.022	0.04	0.046*	0.390	0.02	0.18	0.069	0.09	0.22
**Processing speed**									
Trail Making Test A [s]	− 0.053	0.07	0.010*	− 1.818	0.09	< 10^−3^*	0.038	0.01	0.78
Trail Making Test B [s]	− 0.205	0.11	< 10^−3^*	− 3.644	0.06	0.014*	− 0.502	0.06	0.11
SDMT [Total correct]	0.058	0.05	0.005*	1.086	0.03	0.043	0.215	0.04	0.067
**Memory**									
PAL [first trial score]	0.019	0.03	0.025*	0.197	0.01	0.37	0.090	0.04	0.079
PAL [Total errors]	− 0.045	0.12	0.067	− 0.375	0.11	0.55	− 0.244	0.06	0.15
Word pair learning [total errors]	− 0.025	0.01	0.29	− 0.864	0.02	0.158	− 0.141	0.02	0.31
Word pair recall [total errors]	− 0.015	0.02	0.069	− 0.345	0.02	0.118	− 0.092	0.06	0.049*
**Pattern recognition and spatial working memory**									
PRM [Percent correct]	0.017	0.01	0.44	0.660	0.01	0.25	0.033	0.00	0.78
SRM [Percent correct]	0.056	0.04	0.026*	0.485	0.00	0.46	0.336	0.07	0.017*
SOC [Mean moves]	− 0.008	0.06	0.016*	− 0.101	0.03	0.25	− 0.034	0.05	0.077
SOC [Mean time]	0.017	0.15	0.19	0.374	0.14	0.28	0.046	0.08	0.52
**Longitudinal change in intelligence**									
ΔIQ._65-20_	0.002	0.01	0.27	0.042	0.01	0.31	− 0.006	0.00	0.56
ΔIQ._65-57_	0.001	0.00	0.61	0.038	0.00	0.45	− 0.012	0.03	0.11

To test whether a reduced cerebrovascular response could be a result of brain atrophy, we correlated ΔCBF_Vis.Act_ to the volumes of the segmented brain and to the cortical thickness using a regional surface-based analysis. This analysis yielded no significant correlations between ΔCBF_Vis.Act_ for any regions (*p* > 0.13).

Overall, these observations demonstrate a correlation between reduced cerebrovascular response to neuroactivation and subclinical cognitive deficits and that the reduced ΔCBF_Vis.Act_ was not explained by brain atrophy.

Hereafter, we examined whether a reduced cerebrovascular response affected the metabolic response by correlating ΔCBF_Vis.Act_ to change in lactate concentration from neuroactivation (ΔLac_Vis.Act_). An inability to increase CBF sufficiently in response to neuronal activation could result in exacerbated lactate production. Visual stimulation resulted in a significant (*p* < 10^−6^) increase in lactate concentration of 8.3% (0.060 ± 0.126 mmol/l) in the visual cortex (Fig. 3F); however, we did not observe a significant correlation between ΔLac_Vis.Act_ and ΔCBF_Vis.Act_ (*p* = 0.53, not shown). This observation demonstrates that a low ΔCBF_Vis.Act_ observed in some subjects was not severe enough to affect the metabolic response and lactate production.

Last, we examined whether a reduced ΔCBF_Vis.Act_ could be related to longitudinal changes in intelligence from age 20 (ΔIQ._65–20_) and 57 (ΔIQ._65–57_), but no correlations were observed (see supplementary information Figure S2).

### Cerebrovascular response to breath holding

We examined the global CVR by measuring the change in CBF and CMRO_2_ in response to a breath holding challenge. Breath holding caused a significant (*p* < 10^−6^) increase in CBF of 22.5 ± 7.4% (Fig. 3G). To examine whether CVR was related to cognitive performance, ΔCBF_BH_ and ΔCMRO_2,BH_ were correlated to the performance of the cognitive tests. Reduced ΔCBF_BH_ correlated with poorer performance of IST 2000R, word pair recall and SRM (Table 2). After correction for multiple comparisons, the correlations were no longer significant (*p* > 0.104). ΔCMRO_2,BH_ was not correlated with any cognition parameters (*p* > 0.108). These observations demonstrated a correlation between reduced CVR and reduced general cognition and processing speed. However, the reduction was not severe enough to affect the CMRO_2_ response to breath holding.

Additionally, we examined if reduced CVR could be related to longitudinal changes in 11 from age ~ 20 years (ΔIQ._65–20_) and ~ 57 years (ΔIQ._65–57_), but no significant correlations were observed (see supplementary information Figure S2).

Last, we examined if ΔCBF_Vis.Act_ was correlated with ΔCBF_BH_ to test if the possible vascular deficits affected both measurements of neurovascular function; however, a significant correlation was not observed (*p* = 0.58, not shown). This observation indicates that the subjects can demonstrate impaired cerebrovascular response to neuroactivation but not impaired reactivity to CO_2_-induced vasodilation, and vice versa.

### Resting physiology

To test if resting cerebral physiology was correlated with cognitive function, the gCBF, gCMRO_2_, and NAA were correlated with the cognitive parameters. The gCBF was significantly correlated with SDMT performance (*p* = 0.048), and the NAA concentration was significantly correlated with word pair recall performance (*p* = 0.048). After correcting for multiple comparisons, the correlations were no longer significant (*p* > 0.24). No correlations were observed for the gCMRO_2_ (see supplementary information Table S1).

### Health parameters and lifestyle factors

To test if health parameters or lifestyle factors could be related to impaired cerebrovascular function, we correlated ΔCBF_Vis.Act_ and ΔCBF_BH_ to the health parameters obtained in the study.

In the full linear regression model with all health parameters as regressors, we found a nearly significant correlation between ΔCBF_Vis.Act_ and BMI (*p* = 0.075), but no significant correlation with exercise frequency (*p* = 0.83) or any other parameter (*p* < 0.14). Using stepwise regression, the best model for describing ΔCBF_Vis.Act_ included the parameters alcohol usage, diabetes, hypertension, hypercholesterolemia, and BMI. A model including these parameters resulted in a significant negative correlation with BMI (*p* = 0.034) but a nonsignificant correlation with the other parameters. For ΔCBF_BH_, we observed no correlation with any health parameters in the full regression model. From the stepwise regression, we found that a model with hypertension, exercise frequency, and BMI as regressors was the best description of ΔCBF_BH_; however, no significant correlations were observed.

## Discussion

Impaired cerebrovascular function is observed in both VD and AD [3, 4]. Additionally, the risk factors for AD and VD, such as hypertension, hypercholesterolemia, and diabetes, are similar to those for vascular disease, suggesting an involvement of impaired cerebrovascular function [3, 48, 49]. Recent studies in mouse models have also demonstrated that abnormal tau tangles and aβ protein accumulation disrupt the neurovascular coupling, suggesting a mechanistic correlation [50, 51] between reduced cerebrovascular response to activation and development of age-related cognitive decline.

Patients with dementia have widespread and severe alterations in brain cells, including degeneration of VSMCs, pericyte loss, and endothelial damage. These defects will affect the cerebrovascular response, which increases the difficulty of determining the actual causality between a neurovascular uncoupling and the development of disease when examining patients with manifest cognitive problems and dementia. In the present study, we instead examined a group of subjects aged 64–67 as part of a population cohort without neurodegenerative disease. Using this setup, we demonstrated that cerebrovascular response to neuroactivation and global CVR are also impaired in subjects with only subclinical cognitive deficits and before overt neurodegenerative disease. Impaired cerebrovascular function was independent of brain atrophy. Overall, these results suggest that cerebrovascular function could be a very early sign of age-related declining brain health, with a trajectory pointing towards neurodegeneration.

The neurovascular coupling causing the increase in cerebral perfusion from neuronal activation occurs by a complex pathway with multiple intermediate steps. The release of neurotransmitters from neuroactivation, most notably glutamate, proceeds via a feedforward mechanism to the release of vasoactive agents [15]. Synaptic glutamate triggers N-methyl-d-aspartate (NMDA) receptors, which mediate the influx of Ca^2+^ into neurons. Higher intracellular Ca^2+^ promotes nitric oxide synthase (nNOS) and nitric oxide (NO) production [16]. NO causes vasodilation of the surrounding arteries through the generation of cGMP in VSMCs [52]. Astrocytes also play an important role in neurovascular coupling and mediation of vasodilation [53, 54]. Influx of Ca^2+^ in astrocytes from glutamate activation promotes the release of arachidonic acid and prostaglandins, which both have vasodilating effects. Additionally, higher Ca + in astrocyte end-feet will open K + channels and release K^+^, which has a direct vasodilative effect [55]. At the capillary level, the dilation of the vessel is controlled by pericytes through similar pathways as VSMC control. The cause for impaired cerebrovascular response observed in present study could be an alteration of one or more of these steps. For example, loss of function or reduction of pericytes [48, 56] could result in reduced neurovascular coupling. Pericyte-deficient mice demonstrate reduced neurovascular coupling, among other hemodynamic alterations [57], and the accumulation of aβ proteins interacts with pericytes, thus causing inhibited function or loss of cells [50, 51, 56]. Additionally, human patients with apolipoprotein E4 (APOE4) alleles show accelerated age-related degeneration of pericytes [58]. Abnormal tau protein development could also be a cause for impaired neurovascular coupling, as demonstrated by the correlation between tau protein tangles and neurovascular uncoupling [50].

Neurovascular coupling may also be affected by reduced endothelial function and signaling. Deposition of aβ proteins in the vessel walls of the cerebral arteries causes cerebral amyloid angiopathy (CAA), which directly affects vascular function and neurovascular coupling [59]. Reduced endothelial function can also arise from oxidative stress and the generation of ROS, inhibiting endothelial signaling pathways [3, 60]. ROS production can lead to reduced endothelial eNOS expression and NO availability and reduce the vasodilation pathway [60]. Dysfunction of astrocyte signaling in neurovascular coupling could also be a cause of the inhibited response. The multifaceted signaling between astrocytes and vessels is affected by ageing, particularly with disruption of astrocytic end-feet [48]. Lastly, the reduced cerebrovascular response could also be a result of fewer activated neurons. We attempted to minimize this possibility by calculating the vascular response only in the most activated region, where we would expect full neuronal activation. Also, we did not observe correlation between ΔCBF_Vis.Act_ and atrophy nor cortical thickness which suggests that a smaller CBF response was not a result of fewer available neurons.

Vasodilation from breath holding is caused by the accumulation of CO_2_, which directly causes relaxation of VSMCs. Age-related vessel alterations, such as atherosclerosis and arterial stiffness, impair VSMC function and reduce vasomotor skills [17]. Therefore, the reduced vasodilation in response to breath holding likely reflects the reduced VSMC function and vessel vasotone modulation, perhaps as a result of atherosclerosis.

We did not observe a correlation between the change in lactate concentration from neuroactivation and ΔCBF_Vis.Act_. This finding indicates that the impaired cerebrovascular response seen in subjects with poor cognitive performance was not severe enough to cause an actual metabolic deficit.

We failed to observe any correlations between cerebrovascular function and changes in intelligence from age 20 or 57 years. A reason for this could be that we only measured intelligence and no other cognitive functions at earlier ages and that overall, little change in intelligence over time was observed in the group. This is evident from the distributions of ΔIQ._65–20_ and ΔIQ._65–57_ (see supplementary material Figure S2C-D), where almost all participants were within one standard deviation of change in intelligence, which makes it difficult to examine correlations with age-related decline of intelligence.

Significant correlations between cerebrovascular functions and cognition were observed in many cognitive domains, with largest effect sizes and most significant correlations observed for current intelligence and more a complex measure of processing speed, requiring an executive function aspect (flexibility). The effect sizes were relatively small with the most significant cognitive parameters (IST 2000R and Trail Making Test B) explaining approximately 11–13% of the variance in ΔCBF_Vis.Act_. This demonstrates that the small cognitive deficits are not simply explained by impaired ΔCBF_Vis.Act_ but that other contributing or innate mechanisms also affect cognitive performance.

### Resting metabolism

Resting metabolism did not correlate with cognition. Reduced responsiveness to physiologically stressful situations will likely be affected before resting physiology, which is similar to the disease progression of most cardiovascular diseases. Therefore, the participants in this study likely were not affected to a sufficient degree in order to demonstrate alterations of the resting physiology.

### Health parameters and lifestyle factors

We observed a significant negative correlation between BMI and ΔCBF_Vis.Act_, thus demonstrating an association between a healthy lifestyle and the maintenance of a responsive cerebrovascular function to neuroactivation. Exercise has been shown to preserve cognitive functions in ageing humans [45], and the possible connection could occur through the maintenance of cerebrovascular function in response to neuroactivation.

### Strengths and limitations

The main strength of the study is that we directly assessed change in perfusion by quantitatively measuring the increase in CBF to standardized neuronal activation. The use of ASL to measure quantitative CBF changes from neuroactivation demonstrates similar results as the use of PET imaging based on a study comparing the techniques [26] and has good reproducibility [27]. However, the combined BOLD and ASL MRI measurement also has some limitations. Most notably, when using BOLD imaging to locate the activated brain region, we find the area with the most significant BOLD response. This does not necessarily directly translate to the area with most activated neurons, as the BOLD signal relies on changes in deoxyhemoglobin from elevated CBF and does not directly measure neuronal activity. If the cerebrovascular function is severely impaired in an activated region, the BOLD analysis may not classify this area as part of the most activated region. However, if a participant demonstrates severe cerebrovascular dysfunction, this will also be present in the remaining activated region classified by the BOLD-analysis, and we will still observe a small ΔCBF_Vis.Act_ from this subject.

The ASL techniques also have some limitations. When using the dual-echo sequence, we can only apply a single post labelling delay ASL methodology. This means that changes in arterial arrival times (ATT) could potentially affect the acquired CBF maps. If ATT is significantly delayed for example from stenosis or atherosclerosis, the measured ASL signal would be underestimated. However, we mainly rely on the relative change in the ASL signal during neuroactivation, and we do not expect a large change in ATT between resting state and during neuroactivation reducing potential effects from ATT. Also, none of the participants had a diagnosis of large cerebral artery stenosis or occlusion minimizing the risk for significant delayed ATT.

We failed to measure a significant activation pattern from visual stimulation in some of the participants. In half of these measurements (16 acquisitions), the subject demonstrated excessive motion during the MRI scan, which compromised the data quality and could explain the missing activation. In the remaining subjects, the missing activation likely occurred because the participants were not properly activated, such as by not cooperating or by falling asleep in the scanner. The exclusion of these measurements was based on objective analysis; therefore, we do not expect any biases in relation to cognition to be introduced by the exclusion process.

A further limitation is that the participating subjects were a comparatively homogenous group regarding health parameters, lifestyle factors, and longitudinal cognitive decline, which makes it difficult to establish whether these factors affect cerebrovascular function. However, this homogenous group can also be regarded as a strength because even in this group, we find a robust correlation between reduced ΔCBF_Vis.Act_ and poor cognitive performance.

A last limitation is that only men were included in the study. The original aim of the cohort, from which the participants were recruited, was to explore intergenerational mobility and differential life-chances in men [18]. Data from the cohort was linked with information from the Danish military draft system to obtain cognitive information from when the subjects were approximately 20 years old. The Danish military draft is only mandatory for men, resulting in only men participating in the cohort. Some gender differences related to cerebrovascular functions exist. Studies have demonstrated that elderly women have better CVR to CO_2_ exposure and better cerebral autoregulation compared to men [61, 62]. Whether these differences are due to innate effects or lifestyle factors is not obvious. Perhaps the better CVR seen in women may constitute as a protective mechanism against age-related decline of cerebrovascular function, which should be investigated in future studies.

## Conclusion

Overall, we demonstrate that a subtle reduction in cognitive function is associated with reduced cerebrovascular function, especially reduced cerebrovascular response to neuroactivation. We observed correlations in multiple cognitive domains, indicating an underlying mechanism that affects the whole brain. The reduced cerebrovascular response could not be explained by brain atrophy. The results from this study suggest that inadequate cerebrovascular function is a contributor to age-related cognitive deficits and could potentially be a very early sign and causal factor in the development of neurodegenerative disease.

## Supplementary Information

Below is the link to the electronic supplementary material.

Supplementary file1 (DOCX 1.50 KB)
