# Supplementary material for: Subclinical cognitive deficits are associated with reduced cerebrovascular response to visual stimulation in mid-sixties men
Source: GeroScience. 2022 Jun 1;44(4):1905–23. doi: 10.1007/s11357-022-00596-2 (PMC9617000; doi:10.1007/s11357-022-00596-2)
Supplement: Supplementary file 1 — Supplementary file1 (DOCX 1.50 KB) [file 11357_2022_596_MOESM1_ESM.docx]

**Supplementary Information**

**Subclinical cognitive deficits are associated with reduced cerebrovascular response to visual stimulation in mid-sixties men**

**Mark B. Vestergaard^1^, Ulrich Lindberg^1^, Maria Højberg Knudsen^1^, Olalla Urdanibia-Centelles^2^, Aftab Bakhtiari^1,2^, Erik Lykke Mortensen^3^, Merete Osler^3^, Birgitte Fagerlund^4,5^, Krisztina Benedek^2^, Martin Lauritzen^2,6^, Henrik B.W. Larsson^1,6^.**

1. Functional Imaging Unit, Department of Clinical Physiology, Nuclear Medicine, and PET, Copenhagen University Hospital Rigshospitalet, Glostrup, Denmark.
2. Department of Clinical Neurophysiology, Copenhagen University Hospital Rigshospitalet, Glostrup, Denmark
3. Department of Public Health, University of Copenhagen, Copenhagen, Denmark.
4. Child and Adolescent Mental Health Center, Copenhagen University Hospital, Mental Health Services CPH, Copenhagen, Denmark.,
5. Department of Psychology, Faculty of Social Sciences, University of Copenhagen, Copenhagen, Denmark.
6. Department of Clinical Medicine, Faculty of Health and Medical Science, University of Copenhagen, Denmark

**Corresponding author**: Mark B. Vestergaard, Department of Clinical Physiology, Nuclear Medicine and PET, Rigshospitalet, Valdemar Hansens Vej 1-23, 2600 Glostrup, Denmark. mark.bitsch.vestergaard@regionh.dk

|  |  | **gCBF** | |  | **gCMRO_2_** | |  | **NAA** | |  |  |
| --- | --- | --- | --- | --- | --- | --- | --- | --- | --- | --- | --- |
| **Cognition domain** |  | **β** | **p** |  | **β** | **p** |  | **β** | **p** |  | **R^2^** |
| **General cognition** |  |  |  |  |  |  |  |  |  |  |  |
| IST 2000R |  | -0.115 | 0.34 |  | 0.044 | 0.17 |  | 0.617 | 0.14 |  | 0.04 |
| ACE |  | 0.027 | 0.67 |  | -0.003 | 0.87 |  | 0.413 | 0.064 |  | 0.03 |
| **Processing speed** |  |  |  |  |  |  |  |  |  |  |  |
| Trail making A [s] |  | 0.155 | 0.23 |  | 0.027 | 0.43 |  | -0.392 | 0.38 |  | 0.03 |
| Trail making B [s] |  | 0.297 | 0.44 |  | 0.050 | 0.58 |  | -0.859 | 0.47 |  | 0.02 |
| SDMT [total corrects] |  | -0.207 | 0.048 |  | -0.047 | 0.092 |  | 0.348 | 0.32 |  | 0.08 |
| **Memory** |  |  |  |  |  |  |  |  |  |  |  |
| PAL [First trail score] |  | -0.019 | 0.68 |  | 0.008 | 0.52 |  | 0.026 | 0.87 |  | 0.01 |
| PAL [Total errors] |  | 0.250 | 0.17 |  | -0.052 | 0.27 |  | -0.280 | 0.64 |  | 0.03 |
| Word pair learning [Total errors] |  | 0.019 | 0.87 |  | -0.016 | 0.61 |  | -0.538 | 0.17 |  | 0.02 |
| Word pair recall [total errors] |  | -0.035 | 0.40 |  | 0.005 | 0.67 |  | -0.278 | 0.048 |  | 0.04 |
| **Pattern recognition and spatial working memory** |  |  |  |  |  |  |  |  |  |  |  |
| PRM [Percent correct] |  | -0.111 | 0.28 |  | -0.013 | 0.61 |  | 0.046 | 0.89 |  | 0.02 |
| SRM [Percent correct] |  | 0.021 | 0.87 |  | 0.005 | 0.89 |  | 0.616 | 0.15 |  | 0.02 |
| SOC (Mean choices to correct, 5 moves) |  | -0.009 | 0.64 |  | -0.004 | 0.41 |  | 0.024 | 0.72 |  | 0.01 |
| SOC (Initial thinking time, 5 moves) [s] |  | 0.094 | 0.37 |  | -0.052 | 0.066 |  | 0.122 | 0.73 |  | 0.03 |

**Table S1. Summary of correlations between gCBF, gCMRO_2_, NAA concentration and cognition.** IST 2000R, Intelligenz-Struktur-Test 2000R; ACE, Addenbrooke's Cognitive Examination; SDMT, Symbol Digit Modalities Test; PAL, paired associates learning; RVP, rapid visual information processing; SRM, spatial recognition memory; SOC, Stocking of Cambridge.

**Figure S1. Histograms of the health parameters and lifestyle factors of the participants in the study.**

**Figure S2. Correlations between longitudinal change in intelligence and cerebrovascular response to neuroactivation (ΔCBF_Vis.Act_) and cerebrovascular reactivity (ΔCBF_BH_).** Cognition was tested at age ~20 using the Børge Priers Prøve (BPP) and again at age ~57 and at the time of MRI scanning at age ~65 using the IST-2000R test. Cognition at age ~65 correlated with cognition at age ~20 (A) and at age ~57 (B). The average IST-2000R test score at age 65 (31.7±9.1) was significantly (p=0.016) lower than the score at age 57 (32.7±8.6) but only minorly affected (1.9% reduction).

To change in cognition from earlier age until today was calculated by normalizing the data and subtracting values from age ~20 (ΔIQ._65-20_) (C) and values from age ~57 (ΔIQ._65-57_) (D) with values of present cognition. No significant correlations were observed between ΔIQ._65-20_ and ΔIQ._65-57_ and the cerebrovascular response to neuroactivation (ΔCBF_Vis.Act_) (E) or cerebrovascular reactivity from a breath hold challenge (ΔCBF_BH_) (F).
